# Supplementary material for: Computational Approach for Probing Redox Potential for Iron-Sulfur Clusters in Photosystem I
Source: Biology (Basel). 2022 Feb 24;11(3):362. doi: 10.3390/biology11030362 (PMC8945787; doi:10.3390/biology11030362)
Supplement: Supplementary file 1 [file biology-11-00362-s001.zip › biology-1593025-supplementary.pdf]

Article

Computational Approach for Probing Redox Potential for Iron-Sulfur Clusters in Photosystem I

Fedaa Ali [1,2], Medhat W. Shafaa [1] and Muhamed Amin [3,4,5]*

[1] Medical Biophysics Division, Department of Physics, Faculty of Science, Helwan university, Cairo 11795, Egypt; fali5@vols.utk.edu (F.A.); shafaa@science.helwan.edu.eg (M.W.S.)
[2] Genome science and technology, The University of Tennessee, Knoxville, USA
[3] Department of Sciences, University College Groningen, University of Groningen, Hoendiepskade 23/24, 9718 BG Groningen, The Netherlands
[4] Universiteit Groningen Biomolecular Sciences and Biotechnology Institute, University of Groningen, 9718 BG Groningen, The Netherlands
[5] Department of Physics, City College of New York, City University of New York, NY 10031,USA
* Correspondence: m.a.a.amin@rug.nl

File S1. The optimized structure of F_X and surrounding residues within ~10 Å.

ATOM	1	N	PRO	A	577	6.394	4.624	2.9	1	0	N
ATOM	2	CA	PRO	A	577	4.966	4.793	2.593	1	0	C
ATOM	3	C	PRO	A	577	4.083	3.702	3.23	1	0	C
ATOM	4	O	PRO	A	577	2.84	3.831	3.225	1	0	O
ATOM	5	CB	PRO	A	577	4.88	4.719	1.038	1	0	C
ATOM	6	CG	PRO	A	577	6.224	4.095	0.627	1	0	C
ATOM	7	CD	PRO	A	577	7.188	4.677	1.664	1	0	C
ATOM	15	N	CYS	A	578	4.755	2.662	3.722	1	0	N
ATOM	16	CA	CYS	A	578	4.22	1.383	4.158	1	0	C
ATOM	17	C	CYS	A	578	5.404	0.465	4.449	1	0	C
ATOM	18	O	CYS	A	578	6.563	0.82	4.369	1	0	O
ATOM	19	CB	CYS	A	578	3.267	0.72	3.148	1	0	C
ATOM	20	SG	CYS	A	578	4.09	0.379	1.525	1	0	S
ATOM	27	N	GLY	A	580	4.73	-2.987	3.428	1	0	N
ATOM	28	CA	GLY	A	580	4.309	-4.271	2.898	1	0	C
ATOM	29	C	GLY	A	580	2.893	-4.749	3.274	1	0	C
ATOM	30	O	GLY	A	580	2.274	-4.267	4.241	1	0	O
ATOM	34	N	PRO	A	581	2.371	-5.765	2.549	1	0	N
ATOM	35	CA	PRO	A	581	0.97	-6.197	2.733	1	0	C
ATOM	36	C	PRO	A	581	0.842	-7.048	3.99	1	0	C
ATOM	37	O	PRO	A	581	0.097	-7.999	4.099	1	0	O
ATOM	38	CB	PRO	A	581	0.634	-6.971	1.452	1	0	C
ATOM	39	CG	PRO	A	581	1.998	-7.507	0.987	1	0	C
ATOM	40	CD	PRO	A	581	2.962	-6.365	1.333	1	0	C
ATOM	49	N	THR	A	586	0.067	-2.668	5.97	1	0	N
ATOM	50	CA	THR	A	586	0.818	-1.639	6.701	1	0	C
ATOM	51	C	THR	A	586	0.652	-0.245	6.056	1	0	C
ATOM	52	O	THR	A	586	1.416	0.69	6.329	1	0	O
ATOM	53	CB	THR	A	586	2.328	-1.921	6.907	1	0	C
ATOM	54	CG2	THR	A	586	2.581	-3.182	7.737	1	0	C
ATOM	55	OG1	THR	A	586	3.007	-1.986	5.657	1	0	O
ATOM	63	N	CYS	A	587	-0.397	-0.094	5.223	1	0	N
ATOM	64	CA	CYS	A	587	-0.507	1.101	4.397	1	0	C
ATOM	65	C	CYS	A	587	-0.295	2.395	5.226	1	0	C
ATOM	66	O	CYS	A	587	-1.118	2.821	6.009	1	0	O
ATOM	67	CB	CYS	A	587	-1.89	1.203	3.741	1	0	C
ATOM	68	SG	CYS	A	587	-2.529	-0.289	2.859	1	0	S
ATOM	75	N	ILE	A	724	4.441	5.732	-4.711	1	0	N
ATOM	76	CA	ILE	A	724	3.237	5.62	-3.85	1	0	C
ATOM	77	C	ILE	A	724	3.056	6.925	-3.057	1	0	C
ATOM	78	O	ILE	A	724	1.994	7.458	-2.796	1	0	O
ATOM	79	CB	ILE	A	724	3.296	4.393	-2.886	1	0	C
ATOM	80	CG1	ILE	A	724	3.426	3.077	-3.69	1	0	C
ATOM	81	CG2	ILE	A	724	2.097	4.329	-1.928	1	0	C
ATOM	82	CD1	ILE	A	724	4.123	1.954	-2.915	1	0	C

ATOM	95	N	ARG	A	728	-0.037	10.187	-3.419	1	0	N
ATOM	96	CA	ARG	A	728	-0.672	10.759	-2.228	1	0	C
ATOM	97	C	ARG	A	728	-0.577	12.298	-2.276	1	0	C
ATOM	98	O	ARG	A	728	-0.517	13.015	-1.303	1	0	O
ATOM	99	CB	ARG	A	728	-0.191	10.175	-0.884	1	0	C
ATOM	100	CG	ARG	A	728	-0.589	8.7	-0.726	1	0	C
ATOM	101	CD	ARG	A	728	0.194	7.965	0.366	1	0	C
ATOM	102	NE	ARG	A	728	-0.323	6.6	0.496	1	0	N
ATOM	103	CZ	ARG	A	728	0.264	5.579	1.158	1	0	C
ATOM	104	NH1	ARG	A	728	1.413	5.759	1.809	1	0	N
ATOM	105	NH2	ARG	A	728	-0.358	4.393	1.198	1	0	N
ATOM	120	N	CYS	B	565	-1.578	-6.494	-2.965	1	0	N
ATOM	121	CA	CYS	B	565	-0.523	-5.866	-3.687	1	0	C
ATOM	122	C	CYS	B	565	0.615	-6.824	-3.701	1	0	C
ATOM	123	O	CYS	B	565	0.536	-7.987	-3.352	1	0	O
ATOM	124	CB	CYS	B	565	-0.119	-4.489	-3.149	1	0	C
ATOM	125	SG	CYS	B	565	0.476	-4.599	-1.4	1	0	S
ATOM	132	N	GLY	B	567	3.908	-5.397	-2.186	1	0	N
ATOM	133	CA	GLY	B	567	4.673	-4.42	-1.441	1	0	C
ATOM	134	C	GLY	B	567	5.244	-3.24	-2.237	1	0	C
ATOM	135	O	GLY	B	567	5.063	-3.109	-3.461	1	0	O
ATOM	139	N	PRO	B	568	6.014	-2.379	-1.526	1	0	N
ATOM	140	CA	PRO	B	568	6.587	-1.163	-2.124	1	0	C
ATOM	141	C	PRO	B	568	7.888	-1.535	-2.83	1	0	C
ATOM	142	O	PRO	B	568	8.893	-0.857	-2.83	1	0	O
ATOM	143	CB	PRO	B	568	6.817	-0.228	-0.932	1	0	C
ATOM	144	CG	PRO	B	568	7.127	-1.204	0.215	1	0	C
ATOM	145	CD	PRO	B	568	6.181	-2.382	-0.053	1	0	C
ATOM	154	N	THR	B	573	3.572	-1.397	-5.805	1	0	N
ATOM	155	CA	THR	B	573	2.685	-2.259	-6.587	1	0	C
ATOM	156	C	THR	B	573	1.187	-1.959	-6.378	1	0	C
ATOM	157	O	THR	B	573	0.374	-2.285	-7.251	1	0	O
ATOM	158	CB	THR	B	573	2.943	-3.786	-6.398	1	0	C
ATOM	159	CG2	THR	B	573	4.301	-4.214	-6.961	1	0	C
ATOM	160	OG1	THR	B	573	2.815	-4.175	-5.033	1	0	O
ATOM	168	N	CYS	B	574	0.819	-1.402	-5.202	1	0	N
ATOM	169	CA	CYS	B	574	-0.56	-1.084	-4.855	1	0	C
ATOM	170	C	CYS	B	574	-1.553	-2.104	-5.408	1	0	C
ATOM	171	O	CYS	B	574	-2.739	-1.867	-5.553	1	0	O
ATOM	172	CB	CYS	B	574	-0.982	0.37	-5.123	1	0	C
ATOM	173	SG	CYS	B	574	-0.341	1.614	-3.904	1	0	S
ATOM	180	N	TRP	B	673	-5.584	4.576	4.34	1	0	N
ATOM	181	CA	TRP	B	673	-5.538	3.109	4.248	1	0	C
ATOM	182	C	TRP	B	673	-4.985	2.39	5.507	1	0	C
ATOM	183	O	TRP	B	673	-4.856	2.954	6.571	1	0	O
ATOM	184	CB	TRP	B	673	-4.821	2.681	2.961	1	0	C
ATOM	185	CG	TRP	B	673	-5.432	3.206	1.69	1	0	C
ATOM	186	CD1	TRP	B	673	-6.658	3.79	1.503	1	0	C
ATOM	187	CD2	TRP	B	673	-4.782	3.213	0.399	1	0	C
ATOM	188	CE2	TRP	B	673	-5.683	3.809	-0.531	1	0	C
ATOM	189	CE3	TRP	B	673	-3.509	2.806	-0.052	1	0	C
ATOM	190	NE1	TRP	B	673	-6.834	4.121	0.165	1	0	N
ATOM	191	CZ2	TRP	B	673	-5.344	4.007	-1.877	1	0	C
ATOM	192	CZ3	TRP	B	673	-3.163	3.014	-1.387	1	0	C
ATOM	193	CH2	TRP	B	673	-4.073	3.61	-2.287	1	0	C
ATOM	205	N	ILE	B	708	-6.539	-4.706	3.253	1	0	N
ATOM	206	CA	ILE	B	708	-6.254	-3.843	2.087	1	0	C
ATOM	207	C	ILE	B	708	-7.371	-3.915	1.043	1	0	C
ATOM	208	O	ILE	B	708	-8.092	-2.995	0.704	1	0	O
ATOM	209	CB	ILE	B	708	-4.886	-4.234	1.474	1	0	C
ATOM	210	CG1	ILE	B	708	-3.766	-3.994	2.505	1	0	C
ATOM	211	CG2	ILE	B	708	-4.637	-3.485	0.158	1	0	C
ATOM	212	CD1	ILE	B	708	-2.41	-4.591	2.122	1	0	C
ATOM	225	N	ARG	B	712	-11.055	-1.362	-0.001	1	0	N
ATOM	226	CA	ARG	B	712	-11.401	-0.853	-1.321	1	0	C
ATOM	227	C	ARG	B	712	-12.821	-1.368	-1.623	1	0	C
ATOM	228	O	ARG	B	712	-13.21	-1.753	-2.703	1	0	O
ATOM	229	CB	ARG	B	712	-10.349	-1.114	-2.428	1	0	C
ATOM	230	CG	ARG	B	712	-8.979	-0.519	-2.035	1	0	C

ATOM	231	CD	ARG	B	712	-7.823	-0.761	-3.024	1	0	C
ATOM	232	NE	ARG	B	712	-6.62	-0.035	-2.573	1	0	N
ATOM	233	CZ	ARG	B	712	-5.397	-0.552	-2.305	1	0	C
ATOM	234	NH1	ARG	B	712	-4.96	-1.66	-2.944	1	0	N
ATOM	235	NH2	ARG	B	712	-4.62	0.017	-1.398	1	0	N
HETATM	250	S	SUL	A	3001	0.9	-2.101	1.477	1	0	S
HETATM	251	S	SUL	A	3002	2.022	-1.029	-1.883	1	0	S
HETATM	252	S	SUL	A	3003	-1.668	-1.269	-1.184	1	0	S
HETATM	253	S	SUL	A	3004	0.318	1.427	0.352	1	0	S
HETATM	254	FE	FEC	A	3005	0.027	0.46	-1.879	1	0	Fe
HETATM	255	FE	FEC	A	3006	-0.954	-0.512	1.106	1	0	Fe
HETATM	256	FE	FEC	A	3007	2.108	-0.234	0.427	1	0	Fe
HETATM	257	FE	FEC	A	3008	0.425	-2.347	-0.78	1	0	Fe

File S2. The optimized structure of F_A and surrounding residues within ~10 Å

ATOM	1	N	VAL	C	4	-2.041	8.029	1.538	1	0	N
ATOM	2	CA	VAL	C	4	-2.838	7.562	0.388	1	0	C
ATOM	3	C	VAL	C	4	-2.862	8.62	-0.724	1	0	C
ATOM	4	O	VAL	C	4	-2.679	9.804	-0.53	1	0	O
ATOM	5	CB	VAL	C	4	-4.314	7.135	0.714	1	0	C
ATOM	6	CG1	VAL	C	4	-5.094	6.692	-0.538	1	0	C
ATOM	7	CG2	VAL	C	4	-4.341	6.01	1.761	1	0	C
ATOM	8	N	CYS	C	20	-2.141	-3.745	-4.675	1	0	N
ATOM	9	CA	CYS	C	20	-2.048	-3.612	-3.215	1	0	C
ATOM	10	C	CYS	C	20	-2.448	-4.923	-2.511	1	0	C
ATOM	11	O	CYS	C	20	-3.46	-5.527	-2.873	1	0	O
ATOM	12	CB	CYS	C	20	-3.006	-2.492	-2.765	1	0	C
ATOM	13	SG	CYS	C	20	-3.051	-2.215	-0.935	1	0	S
ATOM	14	N	PRO	C	21	-1.675	-5.379	-1.476	1	0	N
ATOM	15	CA	PRO	C	21	-2.012	-6.625	-0.785	1	0	C
ATOM	16	C	PRO	C	21	-2.89	-6.467	0.476	1	0	C
ATOM	17	O	PRO	C	21	-3.154	-7.467	1.144	1	0	O
ATOM	18	CB	PRO	C	21	-0.634	-7.182	-0.388	1	0	C
ATOM	19	CG	PRO	C	21	0.148	-5.9	-0.059	1	0	C
ATOM	20	CD	PRO	C	21	-0.329	-4.89	-1.117	1	0	C
ATOM	21	N	THR	C	22	-3.298	-5.211	0.796	1	0	N
ATOM	22	CA	THR	C	22	-4.181	-4.972	1.924	1	0	C
ATOM	23	C	THR	C	22	-5.558	-4.511	1.442	1	0	C
ATOM	24	O	THR	C	22	-6.406	-4.04	2.17	1	0	O
ATOM	25	CB	THR	C	22	-3.605	-4.001	2.989	1	0	C
ATOM	26	CG2	THR	C	22	-2.353	-4.549	3.67	1	0	C
ATOM	27	OG1	THR	C	22	-3.392	-2.704	2.447	1	0	O
ATOM	28	N	VAL	C	24	-6.407	-1.4	0.044	1	0	N
ATOM	29	CA	VAL	C	24	-7.091	-0.19	0.528	1	0	C
ATOM	30	C	VAL	C	24	-7.189	0.824	-0.635	1	0	C
ATOM	31	O	VAL	C	24	-7.337	2.039	-0.48	1	0	O
ATOM	32	CB	VAL	C	24	-6.564	0.456	1.842	1	0	C
ATOM	33	CG1	VAL	C	24	-7.597	1.427	2.444	1	0	C
ATOM	34	CG2	VAL	C	24	-6.202	-0.62	2.877	1	0	C
ATOM	35	N	LEU	C	25	-7.136	0.239	-1.862	1	0	N
ATOM	36	CA	LEU	C	25	-7.392	0.97	-3.075	1	0	C
ATOM	37	C	LEU	C	25	-8.678	0.511	-3.743	1	0	C
ATOM	38	O	LEU	C	25	-9.372	-0.411	-3.378	1	0	O
ATOM	39	CB	LEU	C	25	-6.228	0.906	-4.097	1	0	C
ATOM	40	CG	LEU	C	25	-4.876	1.431	-3.576	1	0	C
ATOM	41	CD1	LEU	C	25	-3.791	1.22	-4.644	1	0	C
ATOM	42	CD2	LEU	C	25	-4.937	2.903	-3.141	1	0	C
ATOM	43	N	CYS	C	47	-3.231	0.361	5.202	1	0	N
ATOM	44	CA	CYS	C	47	-1.956	0.636	4.529	1	0	C
ATOM	45	C	CYS	C	47	-0.846	0.456	5.568	1	0	C
ATOM	46	O	CYS	C	47	-0.899	1.017	6.661	1	0	O
ATOM	47	CB	CYS	C	47	-1.881	2.059	3.924	1	0	C
ATOM	48	SG	CYS	C	47	-0.263	2.552	3.154	1	0	S
ATOM	49	N	VAL	C	48	0.199	-0.343	5.185	1	0	N
ATOM	50	CA	VAL	C	48	1.368	-0.534	6.025	1	0	C
ATOM	51	C	VAL	C	48	2.67	0.029	5.419	1	0	C
ATOM	52	O	VAL	C	48	3.762	-0.193	5.937	1	0	O
ATOM	53	CB	VAL	C	48	1.56	-1.992	6.516	1	0	C

ATOM	54	CG1	VAL	C	48	0.384	-2.426	7.4	1	0	C
ATOM	55	CG2	VAL	C	48	1.783	-2.981	5.361	1	0	C
ATOM	56	N	GLY	C	49	2.542	0.811	4.3	1	0	N
ATOM	57	CA	GLY	C	49	3.627	1.677	3.889	1	0	C
ATOM	58	C	GLY	C	49	4.656	1.109	2.907	1	0	C
ATOM	59	O	GLY	C	49	5.598	1.838	2.529	1	0	O
ATOM	60	N	CYS	C	50	4.503	-0.166	2.53	1	0	N
ATOM	61	CA	CYS	C	50	5.483	-1.017	1.857	1	0	C
ATOM	62	C	CYS	C	50	5.85	-0.764	0.37	1	0	C
ATOM	63	O	CYS	C	50	6.782	-1.421	-0.095	1	0	O
ATOM	64	CB	CYS	C	50	5.022	-2.47	2	1	0	C
ATOM	65	SG	CYS	C	50	3.441	-2.827	1.099	1	0	S
ATOM	66	N	LYS	C	51	5.152	0.155	-0.331	1	0	N
ATOM	67	CA	LYS	C	51	5.585	0.673	-1.633	1	0	C
ATOM	68	C	LYS	C	51	5.512	-0.339	-2.799	1	0	C
ATOM	69	O	LYS	C	51	6.444	-0.502	-3.579	1	0	O
ATOM	70	CB	LYS	C	51	6.972	1.336	-1.573	1	0	C
ATOM	71	CG	LYS	C	51	7.024	2.48	-0.553	1	0	C
ATOM	72	CD	LYS	C	51	6.174	3.691	-0.978	1	0	C
ATOM	73	CE	LYS	C	51	5.765	4.621	0.154	1	0	C
ATOM	74	NZ	LYS	C	51	4.72	4.02	1.066	1	0	N1+
ATOM	75	N	ARG	C	52	4.291	-0.929	-2.962	1	0	N
ATOM	76	CA	ARG	C	52	4.005	-1.821	-4.075	1	0	C
ATOM	77	C	ARG	C	52	2.819	-1.348	-4.954	1	0	C
ATOM	78	O	ARG	C	52	2.242	-2.136	-5.699	1	0	O
ATOM	79	CB	ARG	C	52	3.819	-3.272	-3.601	1	0	C
ATOM	80	CG	ARG	C	52	5.133	-3.852	-3.058	1	0	C
ATOM	81	CD	ARG	C	52	4.988	-5.233	-2.416	1	0	C
ATOM	82	NE	ARG	C	52	4.332	-5.143	-1.103	1	0	N
ATOM	83	CZ	ARG	C	52	4.095	-6.184	-0.315	1	0	C
ATOM	84	NH1	ARG	C	52	4.311	-7.444	-0.77	1	0	N1+
ATOM	85	NH2	ARG	C	52	3.705	-6	0.956	1	0	N
ATOM	86	N	CYS	C	53	2.558	-0.015	-4.899	1	0	N
ATOM	87	CA	CYS	C	53	1.424	0.614	-5.549	1	0	C
ATOM	88	C	CYS	C	53	1.878	1.46	-6.741	1	0	C
ATOM	89	O	CYS	C	53	1.204	2.314	-7.269	1	0	O
ATOM	90	CB	CYS	C	53	0.562	1.447	-4.58	1	0	C
ATOM	91	SG	CYS	C	53	1.572	2.514	-3.431	1	0	S
ATOM	92	N	VAL	C	66	2.624	7.487	-1.949	1	0	N
ATOM	93	CA	VAL	C	66	2.3	7.361	-0.488	1	0	C
ATOM	94	C	VAL	C	66	3.539	7.716	0.399	1	0	C
ATOM	95	O	VAL	C	66	4.091	6.97	1.187	1	0	O
ATOM	96	CB	VAL	C	66	1.611	6.043	-0.057	1	0	C
ATOM	97	CG1	VAL	C	66	1.066	6.129	1.381	1	0	C
ATOM	98	CG2	VAL	C	66	0.476	5.663	-1.022	1	0	C
HETATM	99	S	SUL	C	3211	-1.309	1.57	-0.592	1	0	S
HETATM	100	S	SUL	C	3212	2.279	1.519	0.503	1	0	S
HETATM	101	S	SUL	C	3213	-0.177	-1.12	1.758	1	0	S
HETATM	102	S	SUL	C	3214	0.997	-1.169	-1.731	1	0	S
HETATM	103	FE	FEC	C	3221	1.849	-1.018	0.475	1	0	Fe
HETATM	104	FE	FEC	C	3222	-1.166	-0.885	-0.553	1	0	Fe
HETATM	105	FE	FEC	C	3223	0.915	1.281	-1.546	1	0	Fe
HETATM	106	FE	FEC	C	3224	0.035	1.199	1.265	1	0	Fe

File S3. The optimized structure of F_B and surrounding residues within ~10 Å.

ATOM	1	N	ILE	C	6	-0.95	9.067	-1.282	1	0	N
ATOM	2	CA	ILE	C	6	-0.651	8.159	-0.182	1	0	C
ATOM	3	C	ILE	C	6	-1.753	8.269	0.849	1	0	C
ATOM	4	O	ILE	C	6	-2.419	9.281	0.987	1	0	O
ATOM	5	CB	ILE	C	6	0.734	8.493	0.453	1	0	C
ATOM	6	CG1	ILE	C	6	1.84	8.436	-0.62	1	0	C
ATOM	7	CG2	ILE	C	6	1.041	7.589	1.659	1	0	C
ATOM	8	CD1	ILE	C	6	3.146	9.129	-0.214	1	0	C
ATOM	21	N	CYS	C	10	-3.081	3.043	4.418	1	0	N
ATOM	22	CA	CYS	C	10	-1.952	2.128	4.131	1	0	C
ATOM	23	C	CYS	C	10	-1.873	1.112	5.281	1	0	C
ATOM	24	O	CYS	C	10	-2.007	1.49	6.45	1	0	O
ATOM	25	CB	CYS	C	10	-0.614	2.896	4.012	1	0	C

ATOM	26	SG	CYS	C	10	0.859	1.891	3.524	1	0	S
ATOM	31	N	ILE	C	11	-1.614	-0.172	4.923	1	0	N
ATOM	32	CA	ILE	C	11	-1.318	-1.209	5.902	1	0	C
ATOM	33	C	ILE	C	11	0.079	-1.853	5.724	1	0	C
ATOM	34	O	ILE	C	11	0.397	-2.824	6.409	1	0	O
ATOM	35	CB	ILE	C	11	-2.414	-2.302	6.016	1	0	C
ATOM	36	CG1	ILE	C	11	-2.544	-3.136	4.726	1	0	C
ATOM	37	CG2	ILE	C	11	-3.746	-1.679	6.46	1	0	C
ATOM	38	CD1	ILE	C	11	-3.491	-4.336	4.843	1	0	C
ATOM	50	N	GLY	C	12	0.917	-1.284	4.814	1	0	N
ATOM	51	CA	GLY	C	12	2.317	-1.672	4.769	1	0	C
ATOM	52	C	GLY	C	12	2.665	-2.972	4.026	1	0	C
ATOM	53	O	GLY	C	12	3.678	-3.597	4.33	1	0	O
ATOM	57	N	CYS	C	13	1.844	-3.34	3.003	1	0	N
ATOM	58	CA	CYS	C	13	1.992	-4.635	2.349	1	0	C
ATOM	59	C	CYS	C	13	3.078	-4.728	1.25	1	0	C
ATOM	60	O	CYS	C	13	3.474	-5.84	0.902	1	0	O
ATOM	61	CB	CYS	C	13	0.646	-5.123	1.785	1	0	C
ATOM	62	SG	CYS	C	13	0.061	-4.291	0.241	1	0	S
ATOM	67	N	THR	C	14	3.5	-3.557	0.718	1	0	N
ATOM	68	CA	THR	C	14	4.551	-3.381	-0.278	1	0	C
ATOM	69	C	THR	C	14	4.239	-3.931	-1.691	1	0	C
ATOM	70	O	THR	C	14	5.162	-4.333	-2.411	1	0	O
ATOM	71	CB	THR	C	14	5.999	-3.788	0.158	1	0	C
ATOM	72	CG2	THR	C	14	6.257	-3.509	1.633	1	0	C
ATOM	73	OG1	THR	C	14	6.337	-5.14	-0.083	1	0	O
ATOM	81	N	GLN	C	15	2.959	-3.843	-2.138	1	0	N
ATOM	82	CA	GLN	C	15	2.602	-4.321	-3.476	1	0	C
ATOM	83	C	GLN	C	15	2.613	-3.253	-4.591	1	0	C
ATOM	84	O	GLN	C	15	2.76	-3.614	-5.761	1	0	O
ATOM	85	CB	GLN	C	15	1.221	-5.041	-3.496	1	0	C
ATOM	86	CG	GLN	C	15	1.313	-6.575	-3.611	1	0	C
ATOM	87	CD	GLN	C	15	2.171	-7.28	-2.554	1	0	C
ATOM	88	NE2	GLN	C	15	1.86	-6.991	-1.258	1	0	N
ATOM	89	OE1	GLN	C	15	3.047	-8.082	-2.872	1	0	O
ATOM	98	N	CYS	C	16	2.378	-1.959	-4.255	1	0	N
ATOM	99	CA	CYS	C	16	2.147	-0.968	-5.292	1	0	C
ATOM	100	C	CYS	C	16	3.457	-0.461	-5.893	1	0	C
ATOM	101	O	CYS	C	16	3.513	0.155	-6.936	1	0	O
ATOM	102	CB	CYS	C	16	1.304	0.223	-4.808	1	0	C
ATOM	103	SG	CYS	C	16	2.123	1.224	-3.482	1	0	S
ATOM	110	N	MET	C	27	8.93	2.585	-0.404	1	0	N
ATOM	111	CA	MET	C	27	8.512	2.423	0.981	1	0	C
ATOM	112	C	MET	C	27	9.359	3.256	1.918	1	0	C
ATOM	113	O	MET	C	27	10.45	3.718	1.648	1	0	O
ATOM	114	CB	MET	C	27	8.552	0.978	1.562	1	0	C
ATOM	115	CG	MET	C	27	7.809	-0.064	0.719	1	0	C
ATOM	116	SD	MET	C	27	6.078	0.336	0.275	1	0	S
ATOM	117	CE	MET	C	27	5.324	0.539	1.931	1	0	C
ATOM	128	N	ALA	C	39	3.684	3.755	1.694	1	0	N
ATOM	129	CA	ALA	C	39	3.869	3.986	0.287	1	0	C
ATOM	130	C	ALA	C	39	4.378	5.379	0.131	1	0	C
ATOM	131	O	ALA	C	39	4.386	6.224	1.007	1	0	O
ATOM	132	CB	ALA	C	39	2.621	3.821	-0.609	1	0	C
ATOM	139	N	CYS	C	57	-3.266	-2.325	-5.023	1	0	N
ATOM	140	CA	CYS	C	57	-3.532	-2.014	-3.611	1	0	C
ATOM	141	C	CYS	C	57	-4.765	-2.813	-3.18	1	0	C
ATOM	142	O	CYS	C	57	-5.793	-2.781	-3.862	1	0	O
ATOM	143	CB	CYS	C	57	-3.78	-0.512	-3.359	1	0	C
ATOM	144	SG	CYS	C	57	-4.009	-0.064	-1.576	1	0	S
ATOM	150	N	PRO	C	58	-4.686	-3.509	-2.004	1	0	N
ATOM	151	CA	PRO	C	58	-5.799	-4.348	-1.56	1	0	C
ATOM	152	C	PRO	C	58	-6.68	-3.749	-0.446	1	0	C
ATOM	153	O	PRO	C	58	-7.533	-4.462	0.09	1	0	O
ATOM	154	CB	PRO	C	58	-5.088	-5.605	-1.023	1	0	C
ATOM	155	CG	PRO	C	58	-3.839	-5.007	-0.354	1	0	C
ATOM	156	CD	PRO	C	58	-3.428	-3.861	-1.295	1	0	C
ATOM	164	N	THR	C	59	-6.45	-2.466	-0.058	1	0	N
ATOM	165	CA	THR	C	59	-7.311	-1.894	0.952	1	0	C

ATOM	166	C	THR	C	59	-8.665	-1.469	0.398	1	0	C
ATOM	167	O	THR	C	59	-9.05	-1.601	-0.738	1	0	O
ATOM	168	CB	THR	C	59	-6.691	-0.751	1.785	1	0	C
ATOM	169	CG2	THR	C	59	-5.378	-1.157	2.45	1	0	C
ATOM	170	OG1	THR	C	59	-6.556	0.386	0.952	1	0	O
ATOM	179	N	SER	C	63	-5.573	3.109	-1.005	1	0	N
ATOM	180	CA	SER	C	63	-4.83	3.909	-0.012	1	0	C
ATOM	181	C	SER	C	63	-3.809	4.816	-0.737	1	0	C
ATOM	182	O	SER	C	63	-3.058	5.601	-0.148	1	0	O
ATOM	183	CB	SER	C	63	-4.088	3.143	1.097	1	0	C
ATOM	184	OG	SER	C	63	-5.012	2.592	2.048	1	0	O
ATOM	190	N	ILE	C	64	-3.851	4.653	-2.073	1	0	N
ATOM	191	CA	ILE	C	64	-3.067	5.389	-3.017	1	0	C
ATOM	192	C	ILE	C	64	-3.847	5.427	-4.306	1	0	C
ATOM	193	O	ILE	C	64	-4.921	4.892	-4.492	1	0	O
ATOM	194	CB	ILE	C	64	-1.625	4.812	-3.233	1	0	C
ATOM	195	CG1	ILE	C	64	-0.724	5.809	-3.99	1	0	C
ATOM	196	CG2	ILE	C	64	-1.666	3.425	-3.888	1	0	C
ATOM	197	CD1	ILE	C	64	0.77	5.475	-3.901	1	0	C
HETATM	210	S	SUL	C	3311	2.005	-0.426	0.518	1	0	S
HETATM	211	S	SUL	C	3312	-1.652	-0.847	1.544	1	0	S
HETATM	212	S	SUL	C	3313	-0.702	1.98	-0.531	1	0	S
HETATM	213	S	SUL	C	3314	-0.427	-1.462	-2.023	1	0	S
HETATM	214	FE	FEC	C	3321	-1.809	-0.107	-0.716	1	0	Fe
HETATM	215	FE	FEC	C	3322	1.038	0.485	-1.528	1	0	Fe
HETATM	216	FE	FEC	C	3323	0.123	-1.894	0.312	1	0	Fe
HETATM	217	FE	FEC	C	3324	0.143	0.935	1.457	1	0	Fe
